# Improving Generalizability of Spectral Reflectance Reconstruction Using L1-Norm Penalization

**DOI:** 10.3390/s23020689

**Published:** 2023-01-06

**Authors:** Pengpeng Yao, Hochung Wu, John H. Xin

**Affiliations:** 1 Zhuhai Fudan Innovation Institute, Zhuhai 519000, China; 2 School of Fashion and Textile, The Hong Kong Polytechnic University, Hong Kong, China

**Keywords:** multispectral imaging, spectral reflectance, spectral reconstruction, color reproduction

## Abstract

Spectral reflectance reconstruction for multispectral images (such as Weiner estimation) may perform sub-optimally when the object being measured has a texture that is not in the training set. The accuracy of the reconstruction is significantly lower without training samples. We propose an improved reflectance reconstruction method based on L1-norm penalization to solve this issue. Using L1-norm, our method can provide the transformation matrix with the favorable sparse property, which can help to achieve better results when measuring the unseen samples. We verify the proposed method by reconstructing spectral reflection for four types of materials (cotton, paper, polyester, and nylon) captured by a multispectral imaging system. Each of the materials has its texture and there are 204 samples in each of the materials/textures in the experiments. The experimental results show that when the texture is not included in the training dataset, L1-norm can achieve better results compared with existing methods using colorimetric measure (i.e., color difference) and shows consistent accuracy across four kinds of materials.

## 1. Introduction

Spectral reflectance reconstruction in the multispectral imaging system (MIS) has attracted a lot of attention in recent years [1,2,3,4,5,6,7,8]. The objective is to obtain a full spectral reflectance image of the objects (e.g., fabric) such that accurate color reproduction can be performed. Multispectral imaging has its advantage over conventional three-channel color imaging because it can provide the full spectral information in the visible band (i.e., 400 nm–700 nm), which can be used for accurate color measurement [9,10,11]. Applications of MIS also include fruit classification [12], art archiving [13], and color constancy determination [14] among many others. In our study, multispectral imaging refers to using 16 narrowband channels to estimate the full spectral which consists of 31 channels, similarly defined in [2].

**Figure 1:**
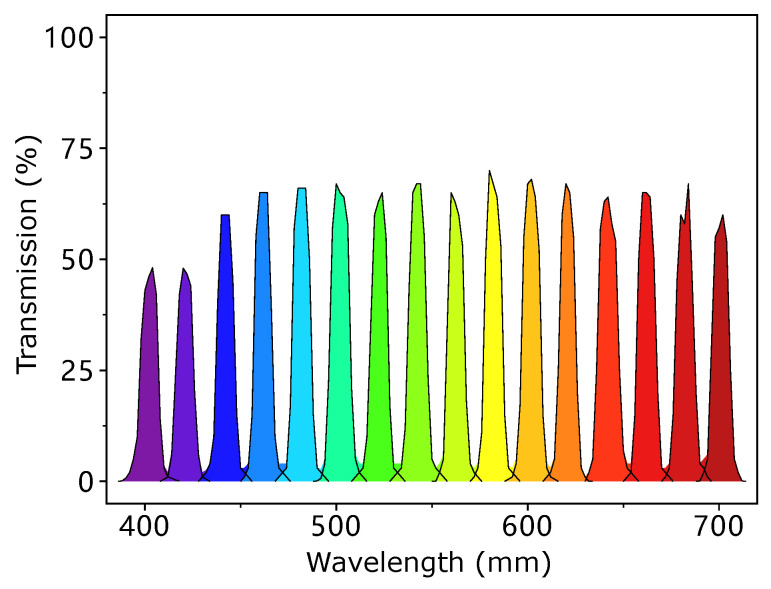
The transmission rate of 16 filters.

In MIS, spectral reflectance reconstruction refers to the process of reconstructing spectral reflectance from the response of multispectral images at different narrow-band wavelengths [3,13]. The transmission rate of a typical set of narrowband filters is shown in Figure 1. In most of the cases, there is a need to find a mathematical mapping to transform a camera’s response vector (with dimension *c*) to a reflectance vector (with dimension *m*), where *c* is less than *m*.

In the literature of multispectral imaging, several reflectance reconstruction techniques have been proposed, including Wiener estimation [3,4,5], Least-square estimation method [6,13,15,16], and Kernel-based methods [7,8]. These methods usually have too many parameters involved in estimating the mathematical mapping between the response and reflectance. Take Pseudo-Inverse as an example, it has m×c parameters, where *m* is the dimension of reflectance vector and *c* is the dimension of response vector. The number of parameters grows linearly with the value of *c*. In color measurement applications [10], it is not uncommon that the number of channels *m* and *c* is as large as 31 and 16 respectively, so the number of parameters will be 31×16=496. Because of a large number of parameters, many training samples are needed for parameter estimation; otherwise, such many parameters may cause overfitting in the reconstruction processing.

In recent years, there has been a lot of work for spectral reconstruction using only 3-channel Red–Green–Blue (RGB) images from off-the-shelf commercial cameras such as Digital Single-Lens Reflex (DSLR) cameras [1,17,18,19,20]. The main methods include regression, sparse coding, and deep neural networks. While the proposed work is also for spectral reconstruction, there is a fundamental difference between our work and the aforementioned recent works, which is the focus on stringent color accuracy. The recent works try to reconstruct 31-dimension spectral data from 3-dimension response data through the massive amount of training samples (each pixel is referred to as a sample while one image can have millions of pixels) with the focus on spectral difference. It is shown that a small spectral difference does not translate to a small color difference [1]. In our work, rather than treating each pixel as a sample, each color patch is treated as a sample. This major difference makes our method not directly comparable to these recent works. Discussion on this issue is further explained in the experiments section.

In this paper, an L1-norm penalization item is added to the Least-square estimation to solve the overfitting issue. The L1-norm item can help the target parameter to achieve sparse property and overcome the overfitting problem in training. Here we take the Pseudo-Inverse as an example, if 5 out of the 16 channels contribute to the final reconstruction results of each reflectance, the number of parameters will decrease from 496 to 31×5=155, which reduces to more than half of the parameters in the Pseudo-Inverse. To verify the results, we prepared four kinds of materials (cotton, paper, polyester, and nylon) with a total of 816 samples. The evaluation results verify the L1-norm penalization method can help to improve the color reproduction accuracy compare to traditional methods.

The paper is organized as follows: Section 2 introduces the basic formulations in spectral reflectance reconstruction; Section 3 presents the current reconstruction algorithms. Section 4 discusses the proposed L1-norm method. Section 5 shows the experiments and then compares the results between our method and other methods. Section 6 and Section 7 discuss the reason why the L1-norm works and reveal the conclusion of our work.

## 2. Formulation of Multispectral Imaging

**Figure 2:**
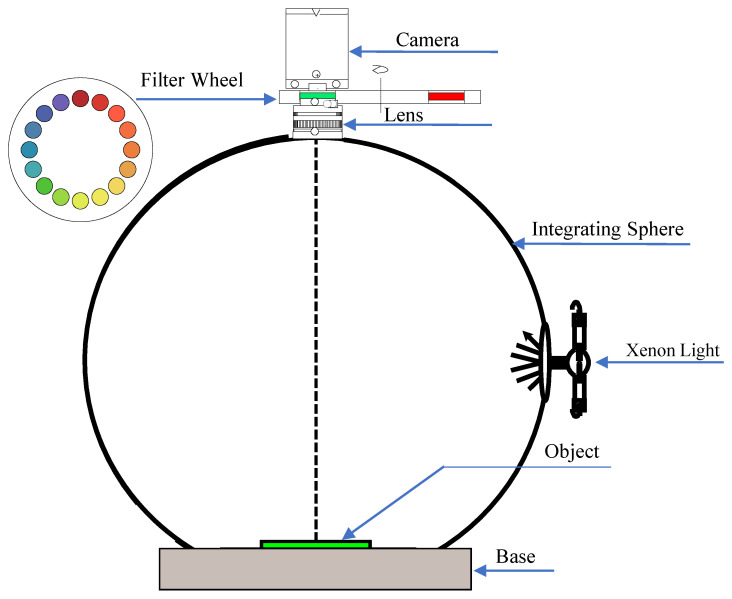
The construction of our MIS.

In our study, a multispectral imaging system is built as illustrated in Figure 2. In the system, a monochrome camera is used for capturing the response images of each narrow-band wavelength $\lambda_{i}\;\left(1\leq i\leq n\right)$ using the corresponding filter in the filter wheel. Narrow-band wavelength filters (transmission rate illustrated in Figure 1) and CCD cameras are commonly used in multispectral systems for color measurement [9,10,21]. The filter wheel with *n* filters is placed between the lens and the camera to filter the light entering the camera. The measured response of the camera is proportional to the intensity of light entering the sensor and we can formulate this as Equation (1). Denote l(λ) to be the spectral power distribution of the imaging illumination, r(λ) to be the spectral reflectance of the samples being imaged, s(λ) to be the sensitivity of CCD camera, $b_{c}$ to be the bias response caused by dark current, and finally, $n_{c}$ to be the noise. In the spectral characterization of the imaging system, spectral sensitivity and bias are recovered by training dataset with known reflectance. Then these responses $u_{c}$ of the *c*th channel can be represented as

(1) $$\begin{array}{cc} u_{c}= & \int l\left(\lambda \right)r\left(\lambda \right)s\left(\lambda \right)d\lambda +b_{c}+n_{c}\\ = & \int m_{c}\left(\lambda \right)r\left(\lambda \right)d\lambda +b_{c}+n_{c}. \end{array}$$

The objective in reflectance reconstruction is to recover r(λ). Note that l(λ) and s(λ) can be merged together into a single term $m_{c}\left(\lambda \right)$ in Equation (1).

In practice, the filters are narrow-band filters, so we can replace the continuous variables with their discrete counterparts and the integral can be replaced to summation. If *N* uniformly spaced samples are used over the visible spectrum, Equation (1) can be rewritten in vector and matrix notation as

(2) u=Mr+b+n

where u is the *c*-dimensional vector of response *u*, and r is a *m*-dimensional vector of reflectance *r*, M is a c×m matrix of spectral responsivity and illumination, b and n are two vector representation of biases and noises respectively.

## 3. Preliminaries

To make this paper self-contained, we briefly summarize the formulations of typical reflectance reconstruction methods, including Least-square estimation (pseudo-inverse method), ridge regression (L2-norm penalization), Wiener estimation, and Kernel methods. Our proposed L1-norm based solution is built based on Least-square estimation, and we will compare our method with all the other methods mentioned in this section.

### 3.1. Least-Square Estimation (Pseudo-Inverse) and Ridge Regression (L2-Norm Penalization)

The subsection provides a brief review of the Least-square estimation method and Ridge Regression [13] while having a detailed discussion of the method. The estimation of reflectance is to find a m×c matrix W that can transform the response u into the estimated reflectance $\hat{r}$,

(3) $$\hat{r}=\mathrm{Wu},$$

A natural thought will be to minimize the difference between the reconstructed $\hat{r}$ and the Wu. So we can formulate the cost function as

(4) $$E=\frac{1}{2}{\left||R-\mathrm{WU}|\right|}_{F}^{2}.$$

In this equation, R is the matrix form of r and U is the matrix form of u. Note that the matrix U in Equation (4) is of size *c* × numberofsamples. The subscript F refers to the Frobenius norm.

Ridge regression can be viewed as adding an L2-norm penalization to Least-square estimation (Equation (4)); the cost function of the ridge can be written down as

(5) $$E=\frac{1}{2}||R-{\mathrm{WU}||}_{F}^{2}+\frac{1}{2}\beta^{2}.$$

The closed-form of solution M can be solved by partially differentiating M in both sides and making it equal to 0. The solution is

(6) $$W={\mathrm{RU}}^{T}{\left({\mathrm{UU}}^{T}+\beta I\right)}^{-1}.$$

When β=0, it is the solution of Least-square estimation.

### 3.2. Wiener Estimation

In Wiener estimation [3], the transform matrix is

(7) $$W_{\mathrm{WE}}={K_{r}M}^{T}{\left({\mathrm{MK}}_{r}M^{T}+K_{n}\right)}^{-1},$$

where $K_{r}$ and $K_{n}$ are the autocorrelation matrices of reflectance and noise, respectively:

(8) $$K_{r}=E\left({\mathrm{rr}}^{T}\right),$$

(9) $$K_{n}=\mathrm{diag}\left\{\sigma_{1}^{2},\sigma_{2}^{2},\cdots ,\sigma_{c}^{2}\right\}.$$

The noise is assumed to be independent across each channel, so the matrix $k_{n}$ is a diagonal matrix in Wiener estimation. The noise $\sigma_{c}$ can be estimated as:

(10) $${\hat{\sigma }}_{c}^{2}=E\left({\left|\right|u_{c}-m_{c}r\left|\right|}_{F}^{2}\right),$$

where $u_{c}$ is the response of the *c*th channel, and $m_{c}$ is the spectral responsivity of the *c*th channel. E denotes the operation of expectation.

### 3.3. Kernel Method

Kernel method is also widely used in spectral reflectance reconstruction [7,8]. It regularizes the Least-square regression in Reproducing Kernel Hibert Space (RKHS). The kernel can be viewed as a function to map the vector in the Least-square method to a new space. Many kernels can be used; in the work [7], the authors applied the Gaussian kernel, Polynomial kernel, Spline kernel, and Duchon kernel.

For example, Gaussian kernel can be defined by

(11) $$k\left(x,z\right)=\exp\left(-\frac{\left||x-z|\right|}{2\gamma^{2}}\right),$$

where γ>0 is a super-parameter. The Gaussian kernel is invariant to rotation and translation, so k(x,z)=k(||x−z||). The corresponding RHKS space is infinite dimensional.

## 4. Proposed Method

In this work, we propose to apply the L1-norm penalized linear regression method for reflectance reconstruction. To the best of our knowledge, it is the first study to use the L1-norm penalized linear regression method for this kind of application. The L1-norm can provide the constrained variable (in our study the constrained variable is W) with sparsity, and this can help to overcome overfitting [22]. The cost function of L1-norm penalized linear regression in reflectance reconstruction is

(12) $$E=\frac{1}{2}||R-{\mathrm{WU}||}_{F}^{2}+{\alpha ||W||}_{1}.$$

In this equation, α is a super-parameter (or a regularization parameter) and can be estimated by cross-validation. W is the weight that transforms response to reflectance. In our work, the response are acquired by placing narrowband filters before the camera, which means that the special reflectance channel is only related to some channels in reflectance. By constraining the weight W, some values of the weight are forced to equal zero. Because the L1-norm is not smooth, we can use the Alternation Direction Method of Multipliers (ADMM) [22] to solve it. A dummy variable can be introduced to Equation (12), and it will be transformed as:

(13) $$\begin{array}{c} W,Z=\arg\min_{W,Z}\frac{1}{2}||R-{\mathrm{WU}||}_{F}^{2}+{\alpha ||Z||}_{1}.\\ s.t\;W=Z \end{array}$$

This is a standard lasso (least absolute shrinkage and selection operator) problem and we can solve it by the following iteration [22].

(14) $$W^{k+1}=\left({\mathrm{RU}}^{T}+\mu Z^{k}-T^{k}\right){\left({\mathrm{UU}}^{T}+\mu I\right)}^{-1}$$

(15) $$Z^{k+1}=\mathrm{soft}\left(W^{k+1}+\frac{T^{k}}{\mu },\frac{\alpha }{\mu }I\right)$$

(16) $$T^{k+1}=T^{k}+\mu \left(Z^{k+1}-W^{k+1}\right)$$

where matrices Z, T are intermediate variables, which can be initialized with zero matrices, and μ should be set larger than zero and I is a unit matrix. The operation soft is a soft-thresholding function as:

(17) soft(u,c)=sign(u)max{|u|−c,0}

Equation (12) can be efficiently solved by using the toolbox in [22]. The pseudo code can be found in Algorithm 1.
**Algorithm 1** L1-norm penalization for spectral reflectance reconstruction1:Initialization: $W^{0},Z^{0},T^{0},k\leftarrow 0$ and μ←0.12:**repeat**3: Update $W^{k+1}$ based on Equation (14)4: Update $Z^{k+1}$ based on Equation (15)5: Update $T^{k+1}$ based on Equation (16)6: k←k+17:**until**$\left|\right|W^{k}\left|-\right|W^{k-1}\left|\right|<\varepsilon$**Output:**$W\leftarrow W^{k}$

## 5. Experiments and Results

### 5.1. Data Preparation

Four kinds of materials are prepared for testing and they are polyester, nylon, paper, and cotton. We use one particular kind of sample (e.g., polyester) as the training set and the remaining three kinds of materials as testing set (i.e., nylon, paper, and cotton). They are selected following Ref. [23]. The objective is to test whether the accuracy of spectral reflectance reconstruction is depending on the type of materials used for training/testing. Each texture includes 204 patches and the reflectance of the color patches was measured using a Spectrophotometer DataColor 650 with an interval of 10 nm. The reason for using the Spectrophotometer is because it is the standard for color measurement [24]. The multispectral images of the 816 samples (4 materials × 204 patches for each material) are acquired by a self-made machine as shown in Figure 2. We use a Xeon lamp and the integral sphere as the illumination light source to make the light more uniform. Moreover, a high-resolution monochromatic camera is employed to capture multispectral images.

**Figure 3:**
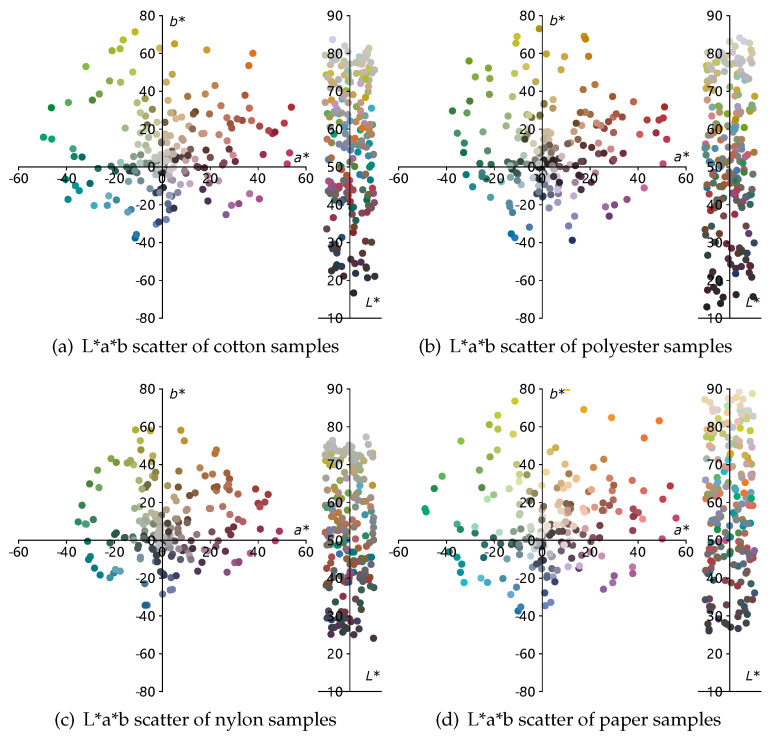
The L*a*b scatter of cotton, polyester, nylon, and paper. The reflectance is measured by DataColor 650 with 10nm interval, gloss include, and 9mm spot size. L*a*b values are computed by computational color tools [25].

The L*a*b space scatters of each texture are shown in Figure 3. The values of these samples are computed by computational color science tools [25]. The reflectance of the samples is in the range of 400–700 nm sampled with 10 nm intervals. The x-axis indicates the wavelength and the y-axis indicates the reflectance measured by the Spectrophotometer.

### 5.2. Evaluation Metric

The color accuracy of the reflectance reconstruction is evaluated both in spectral and colorimetric error. The spectral Root-Mean-Square RMS error between the actual reflectance r and its estimate $\hat{r}$ is calculated as

(18) $$RMS={\left(\frac{{\left(r-\hat{r}\right)}^{T}\left(r-\hat{r}\right)}{m}\right)}^{1/2}$$

where *m* is the dimension of vector *r*. The color difference is evaluated by $\Delta E_{CMC(2:1)}$ [24,25], which is widely used in many industries such as textile and paper production.

### 5.3. Super-Parameter Estimation

**Figure 4:**
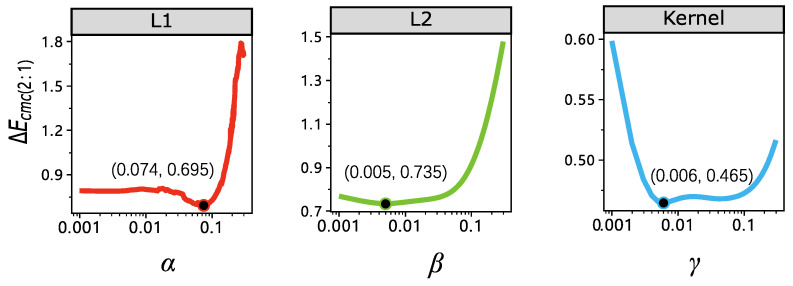
Super-parameter estimation of α, β, and γ in our experiment, the y-axis is the colorimetric difference between the reconstructed spectral and ground truth spectral reflectance. The black point is the minimum point of the ΔE, which means the value we will adopt in the reconstruction.

There are three super-parameters in our experiments that need estimation, the β in Equation (5), the α in the proposed method in Equation (12), and the γ in Equation (11). As an example, γ, α, and β are set to 0.006, 0.074, and 0.0005, respectively, when using cotton as training, which is illustrated in Figure 4. In Figure 4, 70% percent of 204 cotton samples are used for training and the remaining 30% are used for validation. The complete set of values of the super-parameters are listed in Table 1. From the table, the super-parameters are quite stable across different materials.

### 5.4. Results

**Figure 5:**
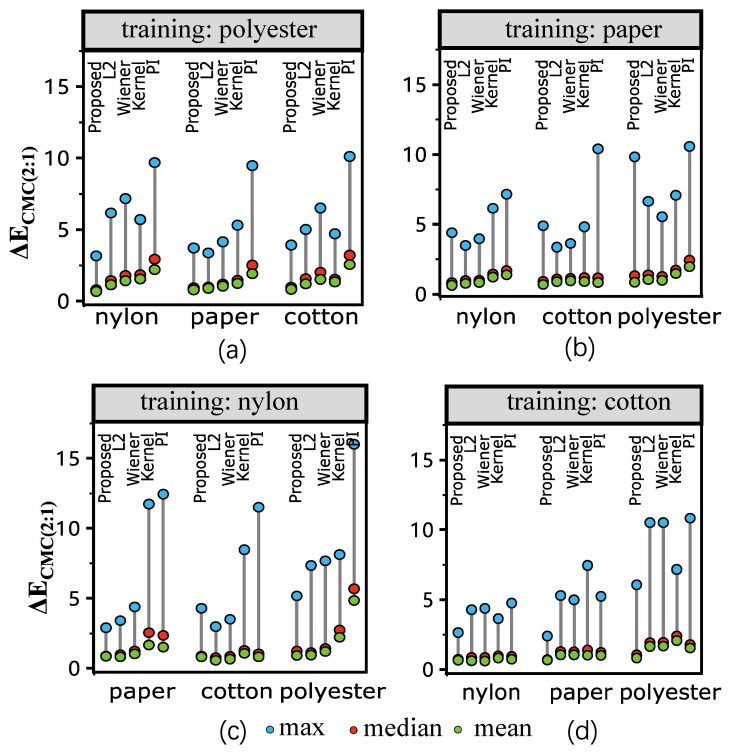
ΔE results under illumination D65 using different materials as training. “Proposed” refers to our L1-norm penalization method, “L2” refers to the ridge regression (L2-norm penalization), “Wiener” refers to the Wiener method and “PI” refers to the least-square estimation (pseudo-inverse method). Sub-figures (**a**–**d**) show the results of using polyester, paper, nylon, and cotton as training samples, respectively. Results show that the proposed method consistently outperforms other methods in the color space; more detailed description is provided in the text.

Figure 5 illustrates the results when one kind of texture (e.g., cotton) is used as a training set (204 samples) and others (204 × 3 samples) as a testing set. The color difference values under D65 are shown in the figure. In Figure 5, the L1-norm method outperforms the Pseudo-Inverse and other estimation methods in all cases when the training set is different from the testing set. The results are consistent when using the mean, the median, and the maximum of the color differences after reflectance reconstruction for the comparison. The mean and median results reveal the overall performance, while the worst-case performance is shown in the maximum color difference results. Specifically, the results of the L1-norm consistently outperform that of the Pseudo-Inverse method using the mean color difference when the training material is different from the testing material. When using the median for the comparison, the L1-norm is better than the pseudo-inverse method in the nylon material for all the testing sets. Similar results are also obtained when using the maximum color difference for comparison in the nylon material. Overall, in the situation when the testing material is unseen (i.e., not present in the testing set), which is often in practice, using L1-norm is better than using Pseudo-Inverse and Wiener estimation for spectral reflectance reconstruction.

**Figure 6:**
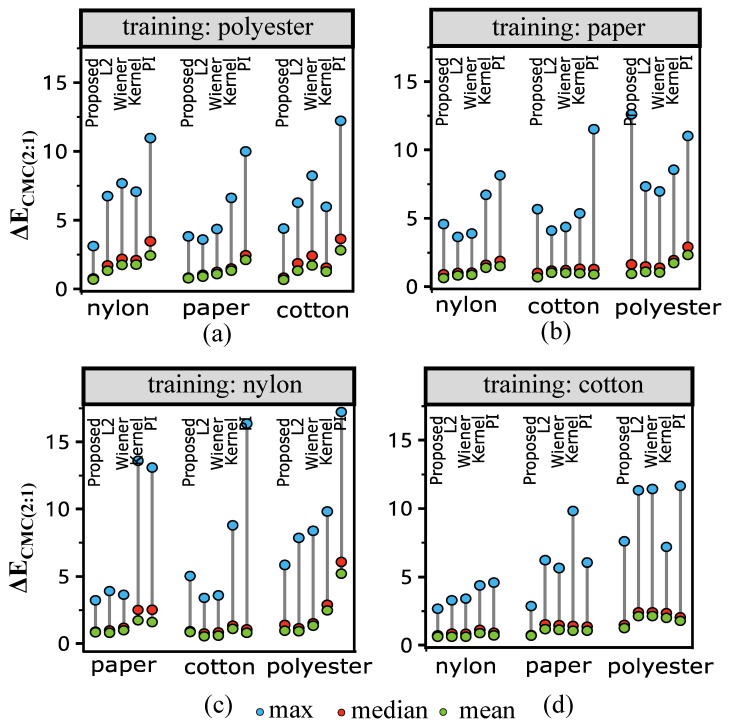
ΔE results under illumination F2 using different materials as training. “Proposed” refers to our L1-norm penalization method, “L2” refers to the ridge regression (L2-norm penalization), “Wiener” refers to the Wiener method, and “PI” refers to the least-square estimation (pseudo-inverse method). Sub-figures (**a**–**d**) show the results of using polyester, paper, nylon, and cotton as training samples, respectively. Results show that the proposed method consistently outperforms other methods in the color space; more detailed description is provided in the text.

**Figure 7:**
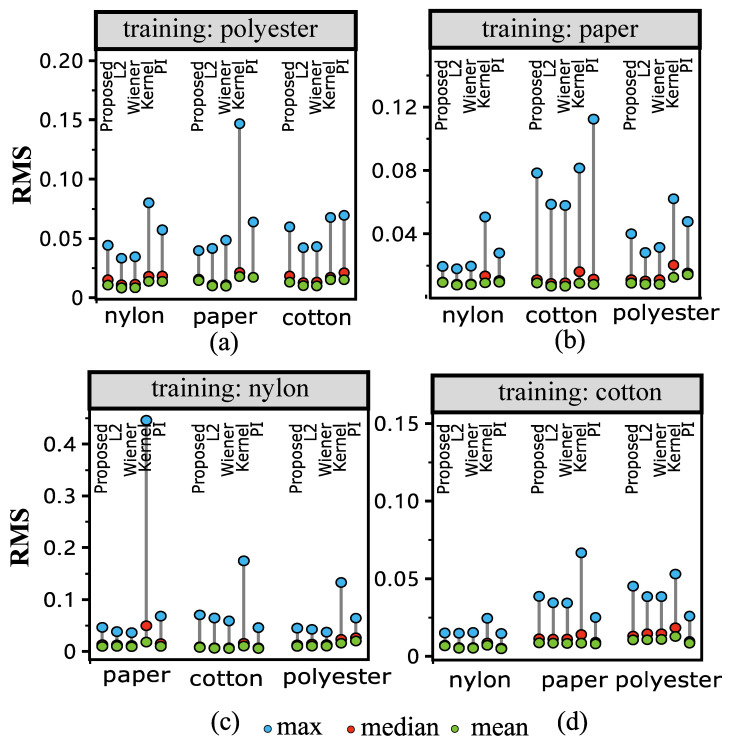
RMS results of spectral reflectance reconstruction using different materials as training. “Proposed” refers to our L1-norm penalization method, “L2” refers to the ridge regression (L2-norm penalization), “Wiener” refers to the Wiener method, and “PI” refers to the least-square estimation (pseudo-inverse method). Sub-figures (**a**–**d**) show the results of using polyester, paper, nylon, and cotton as training samples, respectively. Results show that the proposed method is comparable with other methods in the reflectance space; more detailed description is provided in the text.

Figure 6 shows the values of the color difference with illumination F2. The results tend to be similar to that of Figure 5. Figure 7 shows the spectral difference between the reflectance measured by Spectrophotometer and MIS using RMS, which is not in the color space. From the results, it is interesting to note that the L1-norm method does not show a significant advantage over Pseudo-Inverse and Wiener estimation when using RMS to measure the difference. In practice, color difference is measured in the color space (D65 and F2 in Figure 5 and Figure 6, respectively). This reveals that the L1-norm can be used in situations focused on colorimetry, such as the fabric industry.

**Figure 8:**
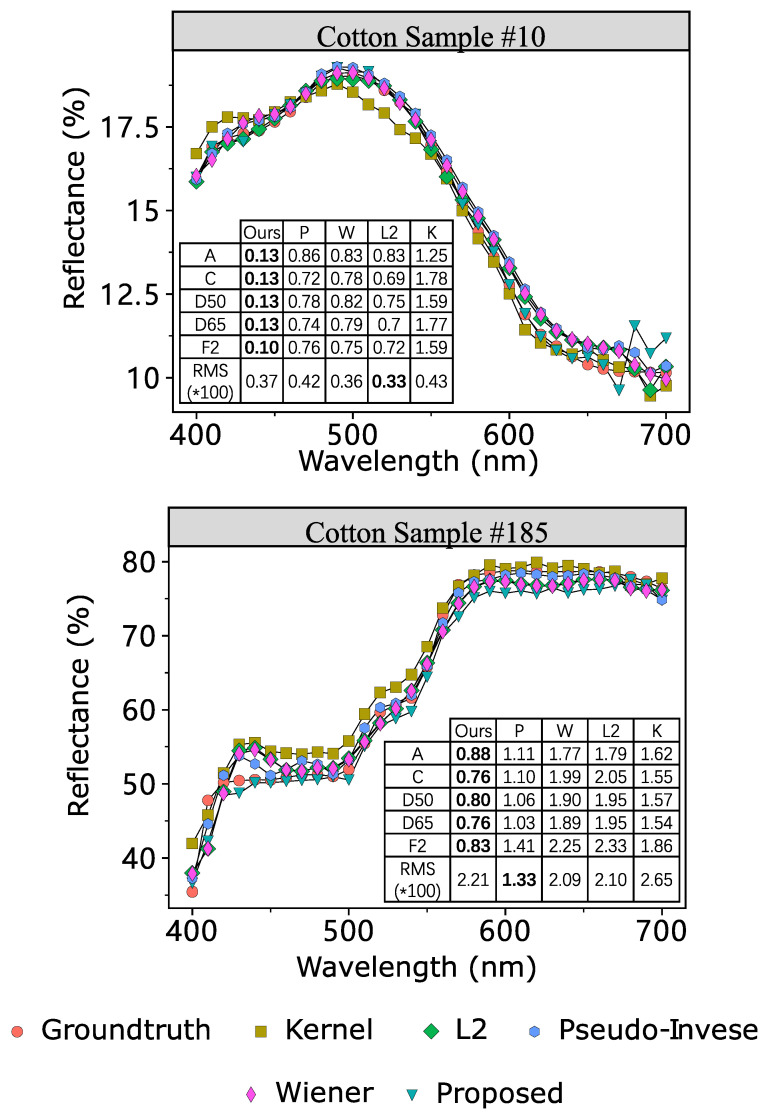
Reflectance reconstruction of a paper sample of the proposed L1-norm estimation and traditional estimations when using cotton for training. Tables inside the plots are the color difference and spectral difference. The A, C, D50, D65, and F2 represent different illumination. The unit of these items is $\Delta E_{\left(CMC(2:1)\right)}$. RMS represents the Root-Mean-Square spectral difference metric. In the tables, method “P” is the briefcase of method Pseudo-Inverse. “Ours” is the proposed method. “W” is Wiener estimation. “K” means kernel method.

Figure 8 is the reconstructed reflectance of two randomly selected cotton samples and the training set used was 204 paper samples. The ground truth is the spectral reflectance measured by a Spectrophotometer and the results are compared with spectral reflectance reconstructed by different algorithms. The accuracy is measured both in color metric and spectral metric. We select five typical illumination sources (i.e., A, C, D50, D65, and F2) and the color difference is measured by $\Delta E_{CMC(2:1)}$. The table in this figure illustrates the color difference and spectral difference individually. In color metrics, the proposed L1-norm method outperforms the traditional methods consistently. However, in spectral metric, the differences of these two samples is still larger than the traditional method. These two samples can verify that the L1-norm penalization can outperform the cut-of-edge algorithm in spectral reflectance reconstruction.

### 5.5. Time Analysis

This section compares the running time of the tested methods in the experiments. All the methods are running on an Intel i7-4790 machine with 32G RAM using Matlab. The training and testing time are listed in Table 2.

When comparing with Ridge Regression (L2) methods and Pseudo-Inverse method, the proposed method requires much more training time; however, they share comparable testing time. As the training can be done offline, the testing time is more important in measuring the efficiency of the methods. From the results, the testing time of the proposed method is comparable to others, except for the Kernel method, which has much worse performance in terms of time analysis.

### 5.6. Comparison with RGB-Based Methods

We also compared three recent RGB-based spectral reconstruction methods, namely, Polynomial methods [18,26], RBF (Radial Basis Function) network method [17], and Gaussian Process method [20]. Sparse coding [1] and deep learning [19] methods require a lot more training samples and they are not included in this work. In this set of experiments, cotton samples are used for training while polyester samples are for testing. The data is first transformed to RGB with D65 as the light source and 1964 observer. The mean color deference is listed in Table 3.

From the results, the average color difference using RGB-based methods is significantly higher than those in Figure 5 which is in the range of 0.4 to 0.9. However, it should be noted that the compassion is unfair as results in Figure 5 are obtained using 16-channel data as input, which contains much more information than the 3-channel RGB data. The results in this sub-section show that recent proposed RGB-based methods cannot be directly applied to reflectance reconstruction tasks with stringent color difference requirements.

## 6. Discussion

This section discusses the reasons for the superior results using the proposed method (i.e., L1 penalization). The advantages of L1 penalization are that (1) it can alleviate the overfitting problem and (2) its sparsity characteristic is more suitable for the underlying spectral reconstruction task.

### 6.1. Overfitting Problem

**Figure 9:**
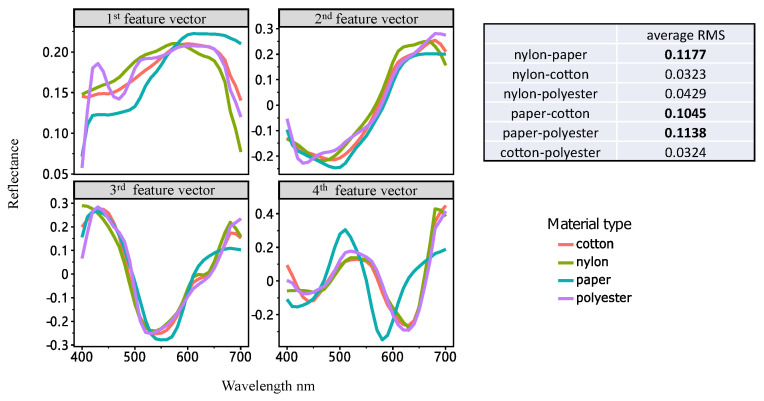
The first 4 feature vectors of 4 kinds of samples (cotton, polyester, nylon, and paper).

Overfitting refers to the problem that a prediction model can only work in the training data but not the testing data [27], as a result, the model is not generalizable (i.e., it works poorly for unseen data). This problem is formed by biased training data (e.g., using a particular material only for training) and lack of penalization term in the model. In order to understand whether the training data is biased, the feature vectors of the four materials used in the experiments are investigated. Each material used in the experiments has its specific property on reflectance [28], which can be shown in Figure 9. Figure 9 plots the first four feature vectors of the four materials. From the figure, one can find that the samples have a great difference especially for the paper samples. When using traditional methods with no penalization term to reconstruct the spectral reflectance, the prediction model tends to work on the (biased) training data only. The L1 penalization method can prevent the model from being dominated by the training data, and thus makes it more generalizable.

### 6.2. Sparsity Characteristic

**Figure 10:**
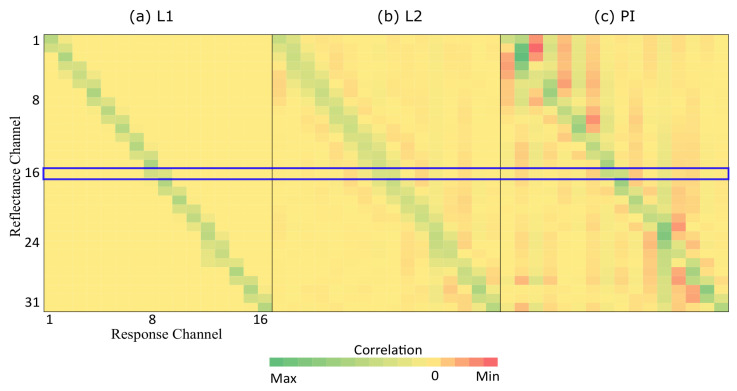
The heatmap representation of weight from different methods.The sub-figures (**a**) show correlation for the proposed L1-norm penalization method, (**b**) shows the L2-norm penalization method, and (**c**) shows the pseudo-inverse method. From the figure, one can see that the correlation is constrained to be zero (yellow color) for most of the entries except the diagonal.

Sparsity refers to the characteristic in the data such that most of the entries (in a vector or matrix) have a value of zero. If the underlying problem (i.e., reflectance reconstruction) possesses the sparsity characteristic, a prediction model which can accommodate the sparse data will perform better than those which cannot. To show that the reflectance reconstruction problem is indeed sparse, Figure 10 shows the correlation between the response (16-channel input) and reflectance (31-channel output) in the form of a heatmap. The sub-figures (a) show correlation for the proposed L1-norm penalization method, (b) shows the L2-norm penalization method, and (c) shows the pseudo-inverse method. From the figure, one can see that the correlation is constrained to be zero (yellow color) for most of the entries except the diagonal.

As we use the narrow-band filters in our system, it is intuitive to consider that the target value (31-channel output) is mostly affected by its neighbourhood channels only, but not channels far away. All the 16 filters are shown in Figure 1. Taking the 450 nm filter as an example, the filter blocks most of the light in the spectral domain, only keeping the light from 430 nm to 470 nm to pass through. So if we reconstruct the reflectance in the 450 nm range in our target results, it should only have a high correlation with the response from 430 nm–470 nm range and have little to no association with the response from the 600 nm range and beyond, which is too far away. By adopting the L1 penalization, the weight of these unrelated channels in the long-distance can be reduced to 0, as shown in the highlighted blue area in Figure 10. However, for the L2-norm penalization (L2) and pseudo-inverse (PI) methods, there are still non-zero correlations (in light red colors), which impact their results.

**Figure 11:**
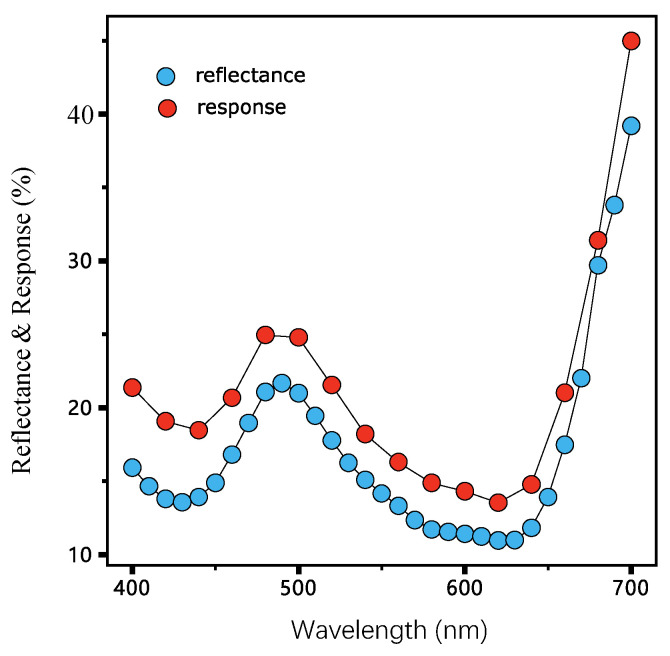
The reflectance and response of a typical cotton sample. The response is a 16-d vector measured by a self-made MIS and reflectance is a 31-d vector measured by a Spectrophotometer.

This sparsity characteristic can also be verified in Figure 11. The reflectance curves measured by the Spectralphotometer and the response curve captured by MIS share a similar shape. It can also be confirmed that the reflectance curve should only be constructed by its neighborhood channels during reflectance reconstruction.

Based on the sparsity characteristic of reflectance, the superior performance of the L1 penalization method is reasonable and understandable. It can inhibit the noise introduced by similar textures and only focus on the accuracy introduced by the MIS.

### 6.3. Material Dependence

From the results of Figure 5 and Figure 6, when polyester and cotton are used as training samples, the result outperforms the others. Meaning, even though L1 penalization improves the generalizability, the results are still sensitive to the training set. In order to verify this, we compare the similarity between the training set (one material, and 204 samples) and testing sets (remaining 3 materials, 612 samples) by KL Divergence using Equation (19).

(19) $$D_{\mathrm{KL}}\left(P\|Q\right)=\sum_{x\in X}P\left(x\right)\log\left(\frac{P(x)}{Q(x)}\right).$$

P(x) and Q(x) here are the distribution of L from Lab color space in training and testing set individually. The results are shown in Table 4. The distances when polyester and cotton are used as training samples are less than that of using paper and nylon. The mean color differences are also smaller.

## 7. Conclusions and Future Work

We propose an L1-norm method for reflectance reconstruction which in certain practical conditions (when the testing texture is unavailable in training samples), the accuracy of the reconstructed reflectance is higher than that using the conventional methods like Pseudo-Inverse and Wiener estimation method. Note that our study is mainly focused on color measurement; therefore, other metrics such as shape-distance sensitivity are not included.

In this paper, we also find a very interesting phenomenon that, while we are optimizing the color difference by spectral domain, the results of the proposed method are better in the color domain. This does not affect the practical application of the proposed method because color difference is measured mainly in the color domain. This phenomenon can be investigated in future work.

## Figures and Tables

**Table 1:** Super-parameters for all the 4 materials as training.

	Cotton	Nylon	Polyester	Paper
λ	0.006	0.008	0.007	0.006
α	0.074	0.082	0.078	0.077
β	0.005	0.003	0.002	0.006

**Table 2:** Training and testing time of compared methods (Unit: ms).

	Proposed	L2	Psudo-Inverse	Wiener	Kernal
Training (20 times)	1759	4	4	1878	6143
Testing (10,000 times)	2232	2230	2231	1473	6,587,500

**Table 3:** Color difference of recent spectral reconstruction methods.

	Polynomial	RBF	Gaussian Process
ΔE	1.80	1.56	2.18

**Table 4:** One material as training and the rest as testing.

	Mean D65 Δ*E*	Mean F2 Δ*E*	KL Diversity
Paper	1.0287	1.1783	1.5252
Polyester	0.8913	0.8189	0.1468
Nylon	1.0013	1.0742	0.281
Cotton	0.8386	0.9897	0.0747

## Data Availability

All data, models, or codes that support the findings of this study are available from the link: https://github.com/tendence/refl_L1.

## References

[1] Lin Y.T., Finlayson G.D. (2019). Exposure invariance in spectral reconstruction from rgb images. Proceedings of the Color and Imaging Conference.

[2] Shen H.L., Cai P.Q., Shao S.J., Xin J.H. (2007). Reflectance reconstruction for multispectral imaging by adaptive Wiener estimation. Opt. Express.

[3] Shen H.L., Xin J.H., Shao S.J. (2007). Improved reflectance reconstruction for multispectral imaging by combining different techniques. Opt. Express.

[4] Shimano N. (2006). Recovery of spectral reflectances of objects being imaged without prior knowledge. IEEE Trans. Image Process..

[5] Murakami Y., Obi T., Yamaguchi M., Ohyama N., Komiya Y. (2001). Spectral reflectance estimation from multi-band image using color chart. Opt. Commun..

[6] Zhang X., Xu H. (2008). Reconstructing spectral reflectance by dividing spectral space and extending the principal components in principal component analysis. JOSA A.

[7] Heikkinen V., Jetsu T., Parkkinen J., Hauta-Kasari M., Jaaskelainen T., Lee S.D. (2007). Regularized learning framework in the estimation of reflectance spectra from camera responses. JOSA A.

[8] Heikkinen V., Lenz R., Jetsu T., Parkkinen J., Hauta-Kasari M., Jääskeläinen T. (2008). Evaluation and unification of some methods for estimating reflectance spectra from RGB images. JOSA A.

[9] Luo L., Shen H.L., Shao S.J., Xin J. (2015). Empirical model for matching spectrophotometric reflectance of yarn windings and multispectral imaging reflectance of single strands of yarns. JOSA A.

[10] Herzog P.G., Hill B. Multispectral imaging and its applications in the textile industry and related fields. Proceedings of the PICS.

[11] Zhang J., Yao P., Wu H., Xin J.H. (2022). Automatic color pattern recognition of multispectral printed fabric images. J. Intell. Manuf..

[12] Jiang J., Gu J. Recovering spectral reflectance under commonly available lighting conditions. Proceedings of the Computer Vision and Pattern Recognition Workshops (CVPRW), 2012 IEEE Computer Society Conference.

[13] Hardeberg J.Y., Schmitt F.J., Brettel H. (2002). Multispectral color image capture using a liquid crystal tunable filter. Opt. Eng..

[14] Mosny M., Funt B. (2006). Multispectral colour constancy. Proceedings of the Color and Imaging Conference.

[15] Hardeberg J.Y. (2001). Acquisition and Reproduction of Color Images: Colorimetric and Multispectral Approaches.

[16] Shen H.L., Xin J.H. (2006). Spectral characterization of a color scanner based on optimized adaptive estimation. JOSA A.

[17] Nguyen R.M., Prasad D.K., Brown M.S. (2014). Training-based spectral reconstruction from a single RGB image. Proceedings of the European Conference on Computer Vision.

[18] Connah D.R., Hardeberg J.Y. (2005). Spectral recovery using polynomial models. Proceedings of the Color Imaging X: Processing, Hardcopy, and Applications.

[19] Shi Z., Chen C., Xiong Z., Liu D., Wu F. Hscnn+: Advanced cnn-based hyperspectral recovery from rgb images. Proceedings of the IEEE Conference on Computer Vision and Pattern Recognition Workshops.

[20] Akhtar N., Mian A. (2018). Hyperspectral recovery from rgb images using gaussian processes. IEEE Trans. Pattern Anal. Mach. Intell..

[21] Zou Z., Shen H.L., Li S., Zhu Y., Xin J.H. (2019). Lighting Deviation Correction for Integrating-Sphere Multispectral Imaging Systems. Sensors.

[22] Boyd S., Parikh N., Chu E., Peleato B., Eckstein J. (2011). Distributed optimization and statistical learning via the alternating direction method of multipliers. Found. Trends Mach. Learn..

[23] Shen H.L., Zhang H.G., Xin J.H., Shao S.J. (2008). Optimal selection of representative colors for spectral reflectance reconstruction in a multispectral imaging system. Appl. Opt..

[24] Schanda J. (2007). Colorimetry: Understanding the CIE System.

[25] Westland S., Ripamonti C., Cheung V. (2012). Computational Colour Science Using MATLAB.

[26] Lin Y.T., Finlayson G.D. (2021). On the Optimization of Regression-Based Spectral Reconstruction. Sensors.

[27] Bishop C.M. (2006). Pattern Recognition and Machine Learning.

[28] Shiradkar R., Shen L., Landon G., Heng Ong S., Tan P. A new perspective on material classification and ink identification. Proceedings of the IEEE Conference on Computer Vision and Pattern Recognition.

